# Supplementary material for: A Pilot Study: Contrasting Genomic Profiles of Lung Adenocarcinoma Between Patients of European and Latin American Ancestry
Source: Int J Mol Sci. 2025 May 19;26(10):4865. doi: 10.3390/ijms26104865 (PMC12111962; doi:10.3390/ijms26104865)
Supplement: Supplementary file 1 [file ijms-26-04865-s001.zip › ijms-3565226-supplementary/Table_S1.pdf]

Table S1. Germline variants identified in our cohort of Mexican patients

Rs ID	Gene	Gene function	REF	ALT	Coding consequence	Change in protein	Clinical significance	
730881673	CDKN2A	Cell cycle kinase	-	T	Insertion	p.Y44fs	P	Hereditary cancer predisposition
200495564	MUTYH	Oxidative DNA damage repair	G	A	Missense	p.R217C	P	Hereditary cancer predisposition
149201802	RAD50	Double Strand Break Repair	C	G	Nonsense	p.Y625*	P	Hereditary cancer predisposition
17580	SERPINA1	Serine protease inhibitor	T	A	Missense	p.E288V	P	Alpha 1 antitrypsin deficiency
121912666	TP53	Tumor suppressor. Guardian of the genome	T	C	Missense	p.Y220C	P	Hereditary cancer predisposition
199907548	CDKN2A	Cell cycle kinase	A	G	Missense	p.I49T	LP	Hereditary cancer predisposition and familial melanoma
925915337	ALK	Receptor Tyrosine Kinase (Insulin Receptor Family)	G	T	Missense	p.H755N	VUS	Hereditary cancer predisposition and neu-roblastoma susceptibility
373153154	ANK2	Membrane proteins and cytoskele-ton protein binding	C	T	Missense	p.S2601L	VUS	Long QT syndrome
144270555	ANK3	Membrane proteins and cytoskele-ton protein binding	T	C	Missense	p.K2306E	VUS	Intellectual disability-hypotonia-spasticity-sleep disorder syndrome
138327406	ATM	Cell cycle kinase	T	G	Missense	p.F1463C	VUS	Hereditary cancer predisposition
150757822	ATM	Cell cycle kinase	A	C	Missense	p.K1992T	VUS	Hereditary cancer predisposition
886041137	AXIN2	Beta-catenin stabiliser	G	A	Missense	p.R465C	VUS	Hereditary cancer predisposition
28997572	BRIP1	Helicase family (interacts with BRCA1)	G	C	Missense	p.I633M	VUS	Hereditary cancer predisposition
184770596	DGUOK	Nucleoside phosphorylation in the mitochondrial matrix.	C	G	Missense	p.P71A	VUS	Mitochondrial DNA depletion syndrome
144228661	FAH	Fumarylacetoacetase (Phenylala-nine catalysis)	A	T	Missense	p.I239F	VUS	Tyrosinaemia type I
139600847	FANCE	Fanconi anemia complementation	C	G	Missense	p.P310R	VUS	Fanconi Anemia
138663330	FANCI	Fanconi anemia complementation	G	T	Missense	p.R1239L	VUS	Fanconi Anemia
17217723	MSH2	DNA mismatch repair	A	G	Missense	p.Y43C	VUS	Predisposición de cáncer hereditario
139134727	MYH9	Non-muscle myosin	G	A	Missense	p.R1466W	VUS	No provided
61756403	POLH	DNA Polymerase Eta	A	G	Missense	p.N233S	VUS	Xeroderma pigmentosum
200264387	RAD50	Double Strand Break Repair	G	A	Intronic	-	VUS	Hereditary cancer predisposition
369560280	RAD50	Double Strand Break Repair	C	T	Missense	p.R725W	VUS	Hereditary cancer predisposition
148716754	RFT1	Intramembrane glycolipid transporte	G	C	Missense	p.S16C	VUS	Congenital glycosylation disorder
1415582429	RUNX1	Core heterodimeric transcription fac-tor	C	T	Missense	p.A329T	VUS	Hereditary haematological cancer
730881980	STK11	Tumor supressor (Serine/Threonine Kinase)	C	T	Missense	p.H202Y	VUS	Hereditary cancer predisposition
758239066	TSC2	Tumor supressor (Growth inhibitory protein)	G	A	Missense	p.S6N	VUS	Hereditary cancer predisposition

VUS: Variant of uncertain significance, **P:** Pathogenic, **LP:** Likely-pathogenic
