# Supplementary material for: A Pilot Study: Contrasting Genomic Profiles of Lung Adenocarcinoma Between Patients of European and Latin American Ancestry
Source: Int J Mol Sci. 2025 May 19;26(10):4865. doi: 10.3390/ijms26104865 (PMC12111962; doi:10.3390/ijms26104865)
Supplement: Supplementary file 1 [file ijms-26-04865-s001.zip › ijms-3565226-supplementary/Table_S2.pdf]

Table S2. Potential clinical relevance of the less studied genes relevant to our cohort.

Gene	Subgroup	Frequency		Therapeutic Target			Biomarker		Ref.
		MX	TCGA	Lung cancer	Other types of cancer	Druggable*	Lung cancer	Other types of cancer	
**RBMX**	Frequent in Mexican cohort (Not-shared)	24%	1.4%	**LUAD:** Depletion of RBMX sensitizes cells to DNA damaging agents.<br>**LC:** Tobacco-induced mutations in RBMX may predispose smokers to develop lung cancer	**Cell lines:** Potential target of cancer therapy (Role in telomere integrity maintenance).<br>Knockdown of RBMX impaired proliferation, migration and invasion.	No		**Esophageal:** Biomarker of poor prognosis (Higher OS in low expression group, p=0.04).<br>**Bladder:** Prognostic biomarker (DFS, p<0.001 and OS, p=0.043, 0.0097 and 0.0099 three datasets)<br>**Various:** Prognostic biomarker and prediction of response to immunotherapy. Higher RR in low expression group.	Tuersun H, 2023, Lui J., 2023, Sheng Y., 2024, Yang Q., 2021, Renieri A., 2014.
**PDE4D**	Frequent in Mexican cohort (Not-shared)	20%	1.2%	**LC:** PDE4 expression (crosstalks with HIF) and promotes progression.<br>Role in development and progression of lung cancer.		Yes	**LUAD:** PDE4D/cAMP/IL-23 axis determines the immunotherapy efficacy.	**Pancreas:** High expression of PDE4D correlates with poor prognosis (Higher OS in low expression group, p=0.002 and RR 3.72 p<0.001)	Liu F., 2019<br>Feng B., 2023<br>Pullamsetti SS., 2013<br>Hsien Lai S., 2020
**JAK2**	Frequent in Mexican cohort (Not-shared)	16%	2.8%	**LUAD:** Overexpression, mutations and amplification of JAK2 participate in cancer progression (proliferation, migration and invasion).<br>Target of multitarget TPX-0005 (Repotrectinib)		Yes	**LUAD:** Mutations associated with Osimertinib resistance.		Xu Y., 2017<br>Wang Z., 2022<br>Ramalingam SS.,2018
**AHI1**	Frequent in Mexican cohort (Not-shared)	16%	1.6%		**CML:** AHI-1 is highly deregulated and mediates TKI-resistance of stem cells	No			Liu X., 2017
**GPR176**	Frequent in Mexican cohort (Not-shared)	16%	1%		**Gastric:** Promotes proliferation, migration, and invasion<br>**Colorectal:** Promotes Cancer progression in vitro and in vivo.	Yes**	**LUAD:** Unfavorable prognostic marker (Expression).	**Gastric:** High expression predicts poor prognosis (Higher OS in low expression group and HR 1.8, p-value 0.003).<br>**Colorectal:** High expression correlates with proliferation and poor overall survival (HR = 2.68, p = 0.023).<br>**Stomach:** Biomarker for prognosis and immune Infiltration (Higher OS in low expression group)	Zhang Y., 2023<br>Ni L., 2023<br>Tang J., 2023<br>Uhlen M., 2017<br>The Human Protein Atlas
**SLC36A4**	Differences in frequency (Shared)	20%	1%		**General:** Immunosuppressive tumor microenvironment mediated by kynurenine transport through SLC36A4.<br>Role in migration. Transporters stand out as potential targets.<br>**Cell lines:** Critical for mTORC1-regulated growth.	No	**LUAD:** Unfavorable prognostic marker (Expression).	**Colon:** High expression of SLC36A4 predicts poor relapse-free survival (p=0.005)	Chen L., 2023<br>Fan S-J., 2016<br>Goberdhan DCI., 2010<br>The Human Protein Atlas
**AP1S1**	Differences in frequency (Shared)	8%	0.2%	**LUAD:** Inhibition of AHI1 increases sensibility in resistant cells to EGFR-TKIs and causes degradation of EGFR.		No			Jeong J., 2023
**NRCAM**	Frequent (Shared)	24%	7.5%		**Liver:** High expression in cancer stem cells.<br>Promotes migration and Epithelial-Mesenchymal Transition	No	**LUAD:** Differential expression between LUSC and LUAD. High expression in LUSC and Low in LUAD.<br>**LC:** Hypermethylated	**Gastric:** High expression is associated with poor prognosis.<br>**Liver:** High expression was associated with a poorer survival (p=0.001)	Bai C., 2022<br>Zhou L., 2023<br>Lucchetta M., 2019<br>Ansari J., 2016

Gene	Subgroup	Frequency		Therapeutic Target			Biomarker		Ref.
		MX	TCGA	Lung cancer	Other types of cancer	Druggable*	Lung cancer	Other types of cancer	
**TET1**	Frequent (Shared)	20%	4.2%	**LC:** Proposed as oncogene. Gain function via loss of TP53. Synergistic with cytotoxic drugs. *K*nockdown confers resistance to EGFR TKIs	**TNBC:** Role in self-renewal of cancer stem cells. **Thyroid:** TET1 promotes oncogenesis and cancer progression (in hypoxia).	Yes**	**LC:** Predictive biomarker for immune checkpoint blockade. **NSCLC:** Downregulation of TET1 was correlated with poor clinical features. Mut-TET1 shows a protective effect: PFS, HR =0.46, p= 0.008 and OS, HR=0.47, p=0.019 **LUAD:** Mutations co-occur with KRAS and predicts poor survival. Higher OS in low expression group (p=0.048)	**Thyroid:** Expression is associated with poor survival (switch-hypoxia).	Yang Q., 2023 Ghazimoradi MH., 2024 Filipczak PT., 2019 Alrahali AA., 2023 Forloni M., 2016 Wu H-X., 2019 Xu Q., 2022

LC: lung cancer, NSCLC: Non-small cells lung cancer, LUAD: Lung adenocarcinoma, CML: Chronic myeloid leukemia, TNBC: Triple negative breast cancer, RR: Response rate, OS: Overall survival, HR: Hazard ratio, PFS: Progression Free Survival, DFS: Disease Free Survival.

* According to DGIbd, ChEMBL and Therapeutic Targets Database.

** Only ChEMBL
